# From mechanisms to precision medicine: the role of organoids in studying the gut microbiota-tumor microenvironment axis

**DOI:** 10.3389/fmicb.2025.1669482

**Published:** 2025-10-13

**Authors:** Si-yang Zheng, You-yu Su, Fu-liang Cai, Da-fang Xu, Yong-qiang Xu

**Affiliations:** Department of Proctology, The First Affiliated Hospital of Huzhou University, Huzhou, Zhejiang, China

**Keywords:** organoids, gut microbiota, host–microbe interactions, tumor microenvironment, microbial metabolites

## Abstract

Intestinal organoids are three-dimensional *in vitro* models derived from patient-specific tissues, which can recapitulate the structural and functional characteristics of the native intestinal epithelium, including interactions with the gut microbiota. In the study of host-microbiota crosstalk within the context of the Tumor Microenvironment (TME), they have become highly effective tools, providing an opportunity to explore the role of microorganisms in carcinogenic processes, immune regulation, and therapeutic responses. Although organoids can successfully simulate key aspects of the TME, certain features—such as systemic immune interactions, neuroendocrine axes, and dynamic microbial communities—remain difficult to fully replicate. This review primarily covers the advances in organoids applied to the research of the microbiota-TME axis, examines their current limitations, and further advocates for their integration with multi-omics and organ-on-a-chip technologies to enhance physiological relevance and the value of translational applications.

## 1 Introduction

### 1.1 The significance of host-microbiome interactions in the TME

Cancer remains a major global health burden, and a central challenge in therapy development is the significant disparity between existing models and the actual TME in human patients. The interaction between the host and the gut microbiota within the TME, a pivotal driver of cancer progression and therapy, has garnered widespread attention in recent years. The tumor microbiome not only significantly influences tumor initiation, progression, and the development of drug resistance (Pich et al., 2025; Qiao et al., 2022) but also plays a crucial role within the TME by modulating immune responses and treatment efficacy (Zhang S. et al., 2025; Grzywa et al., 2017). Advancements in this field have been hampered by the inadequate physiological relevance of conventional models (Williamson et al., 2018), which fail to accurately recapitulate the complex host-microbiota interactions and the dynamics of the TME (Qu et al., 2021; Chen et al., 2024). Within this context, organoids have emerged as a transformative innovation for cancer gut microbiome research. As a novel three-dimensional (3D) *in vitro* model derived from primary tissues, organoids can highly recapitulate the heterogeneity, architecture, and functional characteristics of tumors (Tardito et al., 2024) while also incorporating relevant elements of the gut microbiota, thereby uniquely facilitating cancer-specific investigations. A defining attribute for cancer applications is the ability of organoids to be coupled with patient-derived samples to establish co-culture systems encompassing epithelial cells, stromal fibroblasts, and microbes. This allows for precise dissection of host-microbiota interactions in the cancer immune microenvironment, for instance, through microinjection or microfluidic techniques (Penarete-Acosta et al., 2024) that enable exposure of tumor cells to gut microbiota. This integration not only revolutionizes the experimental platform for studying the tumor microbiome, enabling investigations into inflammation, carcinogenic mechanisms, and personalized treatment responses, but also provides unprecedented insights into precision intervention strategies for cancer-associated microecology. Thereby, it bridges critical knowledge gaps regarding the gut microbiota in tumor biology and propels cancer research towards new heights of clinical translation. We propose that current research has established host–microbe interactions as the core regulatory axis of the TME, with particularly substantial evidence in the contexts of immunometabolic reprogramming and therapeutic response prediction. The core limitations include insufficient depth in mechanistic understanding, delayed advancement in intratumoral microbiota research, and bottlenecks in clinical translation. This field urgently requires the integration of high-resolution technologies (single-cell sequencing + spatial transcriptomics) with biomimetic models (organoids/organ-on-a-chip) to translate phenotypic correlations into mechanisms of causality, while driving intratumoral microbiota research from the stage of descriptive analysis toward that of functional validation (see Table 1).

**Table 1:** Types of organoids and their applications.

Model type	Key features	Advantages	Limitations	Primary applications
Patient-derived organoids (PDOs)	Retain genetic and phenotypic heterogeneity of original tumor; support co-culture with microbiota	High clinical relevance; suitable for personalized drug screening and biobanking	Limited TME (e.g., lack of immune/stromal components); high cost	Individualized drug sensitivity testing; studies of tumor heterogeneity and carcinogenic mechanisms
Adult stem cell-derived organoids	Form 3D structures with crypt-villus architecture; include multiple epithelial cell subtypes	Model normal intestinal development and host–microbe interactions; reproducible	Limited ability to simulate tumor-specific mutations and complex TME dynamics	Research on intestinal barrier function; microbial colonization; inflammation and repair mechanisms
Multi-cell/microbe co-culture organoids	Incorporate immune cells, microbiota, or microbial metabolites (e.g., SCFAs, bile acids)	Better mimicry of in vivo microbial-immune crosstalk; applicable for immunotherapy research	Technically complex; microbial community stability challenging to maintain long-term	Immune regulation studies; immunotherapy response mechanisms; microbial metabolite function evaluation
Organ-on-a-chip integrated models	Combine microfluidics with organoids to simulate fluid flow, mechanical forces, and microbial gradients	Dynamic and spatially controlled culture; real-time monitoring and high-throughput potential	High technical threshold; not yet widely adopted; relatively expensive	Simulating peristalsis and metabolic gradients; bacterial invasion and host response; drug toxicity/pharmacokinetics
Cancer cell line-derived organoids	Established from traditional CRC cell lines (e.g., HCT-116, Caco-2); form 3D structures in Matrigel	Easy culture and genetic manipulation; suitable for high-throughput screening	Lack patient specificity; genetic drift over time; unable to fully replicate TME complexity	Preliminary drug screening; studies of signaling pathways (e.g., Wnt/NF-κB); mechanistic research

## 2 Introduction to and classification of intestinal organoids

Intestinal organoids are three-dimensional (3D) (Qu et al., 2021), self-organizing micro-organ structures that are generated *in vitro* by harnessing the regenerative capacity of stem cells. They can highly recapitulate the complex composition and physiological architecture of the native intestinal tissue. Their core structure is formed by a polarized epithelial layer (Chen et al., 2024) that encompasses a variety of functionally differentiated cell subtypes, such as enterocytes (Moll et al., 2022; Pérez-González et al., 2021). These cells organize into crypt-like folds and cellular compartments in three dimensions, mimicking the crypt-villus unit patterning of the intestine (Pérez-González et al., 2021). The organoid structure also includes a supportive mesenchymal cell layer (Chen et al., 2024), and specific models may incorporate additional elements, such as microvascular fragments, to simulate vascular components. The culture conditions profoundly influence the architecture. When cultured within a laminin-rich Matrigel matrix, the organoids exhibit the correct basal-out polarity. Removal of Matrigel can induce a reversal of polarity to an apical-out configuration (Yavitt et al., 2022; Hirota et al., 2021; Yavitt et al., 2023). These 3D structures form through a self-organizing process and can display intestinal cellular diversity, mechanical properties, and functional behaviors, thereby providing a physiologically relevant model for studying intestinal development and disease.

The colorectal cancer (CRC) cell lines refer to populations of cancer cells isolated from human colorectal cancer tissues or metastatic sites (e.g., lymph nodes) that can be cultured long-term *in vitro* (Luk et al., 2025; Codrich et al., 2021). As essential models in cancer research, their primary principle lies in retaining the molecular characteristics of the primary tumor, thereby laying the groundwork for simulating tumor properties under controlled conditions. Their core advantages include ease of standardized culture, facilitating genetic manipulation, and high-throughput drug screening, which significantly accelerate research into the mechanisms of colorectal carcinogenesis, drug resistance, and the identification of novel therapeutic targets. For instance Somarelli et al. (2020) identified dual CDK2/9 inhibition as a novel combination therapy for colorectal cancer, and Tran et al. (2022) demonstrated the efficacy of BRAF mutation or VEGFR inhibitors in specific cell lines. These models are particularly applied in cancer research to: (1) dissect tumor signaling pathways (Sugito et al., 2022); (2) investigate cancer stem cell properties (Ying et al., 2023; Grigoreva et al., 2025); (3) assess interactions within the immune microenvironment (Teng et al., 2024); (4) develop therapeutic strategies (Zhang S. et al., 2024; Ferreira et al., 2024; Ji et al., 2021); including the validation of tumor-suppressive effects by non-coding RNAs or natural compounds. Notable limitations include: (1) the absence of the complex TME (Castro et al., 2021), limiting their ability to fully recapitulate *in vivo* conditions; (2) potential genetic drift during long-term passaging (Luk et al., 2025; Codrich et al., 2021; Przybylla et al., 2023), leading to a loss of the original tumor heterogeneity; (3) the loss of critical molecular characteristics in some cell lines (Shi et al., 2024) due to mechanisms such as methylation; Substantial biological differences exist among various cell lines, necessitating careful selection of the most appropriate model for specific research questions.

**Figure 1:**
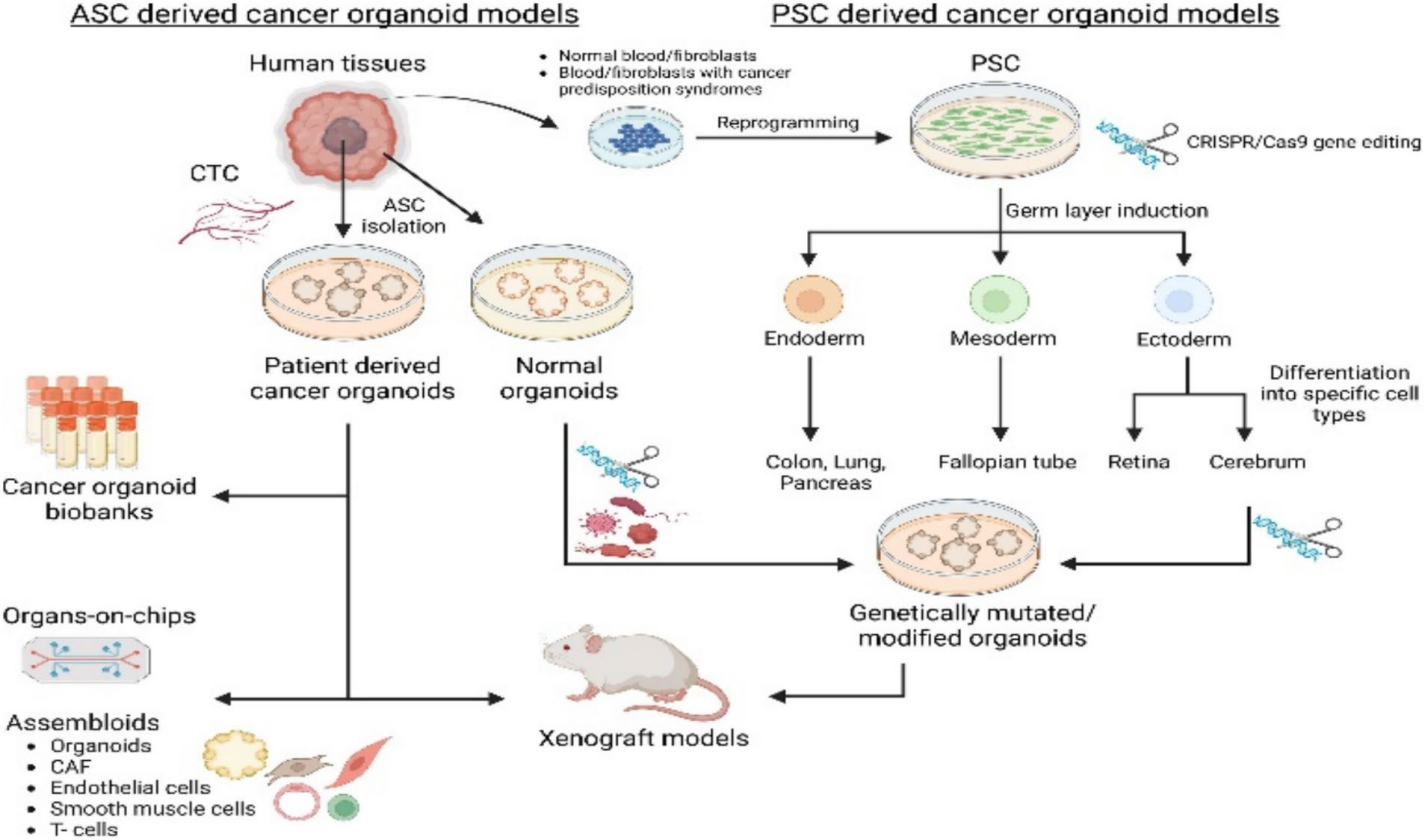
Cancer organoids, derived from either adult stem cells (ASCs) or pluripotent stem cells (PSCs) and incorporating components like circulating tumor cells (CTCs) or cancer-associated fibroblasts (CAFs), are established to model various cancer types and for research applications. Adapted with permission from Yan et al. (2023). Copyright 2023 Cell Stem Cell.

Adult stem cell-derived colorectal cancer organoids (Nakano et al., 2025; Driehuis et al., 2020) are three-dimensional miniature tumor models generated *in vitro* from adult stem cells isolated from patient intestinal tissues (e.g., Lgr5-positive stem cells residing at the crypt base). Their generation leverages the self-renewal and multilineage differentiation capabilities of adult stem cells. By reconstituting a physiological microenvironment *in vitro* (e.g., providing Wnt-signaling niche factors) (Yan et al., 2023), these cells self-organize into organoids that retain the genetic features, cellular heterogeneity, and pathological architecture of the primary tumor (Xu H. et al., 2022). Major advantages include: (1) high preservation of patient tumor’s molecular characteristics and physiological relevance, addressing the limitation of conventional 2D cell lines in modeling tumor heterogeneity (Jin et al., 2025; Wang R. et al., 2022); (2) capability for long-term expansion, enabling the establishment of living tumor biobanks (Mao et al., 2024) (3) facilitation of high-throughput drug screening and personalized therapy evaluation, such as testing chemotherapeutic drug sensitivity (Mao et al., 2024) and developing treatment strategies targeting cancer stem cells (Yan et al., 2023) (e.g., inhibiting inflammatory factors that maintain tumor stemness). Core applications focus on colorectal cancer research: modeling tumorigenesis, investigating specific characteristics of the TME, screening anticancer drugs, and developing effective regenerative medicine strategies. Main limitations include the current absence of non-epithelial components in these models, which impedes research on microenvironmental interactions, and the need for further optimization of standardized culture systems (Figure 1).

Pluripotent stem cell (PSC)-derived intestinal cancer organoids are generated through the differentiation of pluripotent stem cells (such as induced pluripotent stem cells [iPSCs] or embryonic stem cells [ESCs]) to form three-dimensional micro-organ structures (Xu H. et al., 2022), These structures mimic the cellular composition, tissue architecture, and functional characteristics of human intestinal carcinogenesis, thereby providing a highly physiologically relevant *in vitro* tumor model; The principle relies on the plasticity of pluripotent stem cells (Wuputra et al., 2021), utilizing directed differentiation (e.g., first inducing definitive endoderm, then further developing into intestinal tissue to reconstruct the intestinal epithelium) (Matkovic Leko et al., 2023), and can be combined with oncogene introduction (e.g., KRAS) or carcinogen treatment (e.g., Azoxymethane) to induce a cancer-like state, thereby elucidating molecular mechanisms of tumor growth (Huang et al., 2021). Its advantages include the precise modeling of tumor heterogeneity and genetic characteristics, providing an efficient platform for drug screening and personalized therapy evaluation with relatively low cost and short time requirements; Limitations include difficulties in modeling non-epithelial components (e.g., immune cells or TME) (Farin et al., 2023), the technical complexity of 3D culture systems that restricts clinical applications (e.g., regenerative medicine) (Zhu et al., 2023), and significant challenges remain in their development as clinical diagnostic or therapeutic tools; In cancer research, such organoids are widely used in colorectal cancer studies to investigate molecular mechanisms of tumor initiation and progression, conduct large-scale drug testing (predicting patient response), stablish biobanks for basic research, and advance the development of precision medicine strategies (Figure 1).

Gut-on-a-chip systems simulate the structure and function of the human intestine by engineering controllable microenvironments within microchips (Özkan et al., 2024), with a particular focus on replicating the three-dimensional morphology of the intestinal epithelium, fluid flow, and mechanical stresses to investigate physiological and pathological mechanisms, The operating principle involves employing microfluidic channels and cell culture techniques with human cells, coupled with precise control of biochemical factors (Xian et al., 2023; Xiang et al., 2020), to recapitulate processes such as intestinal barrier function, nutrient absorption, and pathogen invasion. Furthermore, through interconnection with other organ chips (e.g., liver-chip) (Milani et al., 2022), they model cross-organ signaling. Advantages include a more accurate mimicry of human intestinal physiology, reduced interspecies variation, and higher physiological relevance compared to conventional two-dimensional cultures; they demonstrate improved cost-effectiveness, encounter fewer ethical concerns, and can be applied to drug screening and disease modeling, Limitations of these systems include high manufacturing costs, challenges in model standardization, and difficulties in replicating complex microenvironments such as microbiome interactions, which hinder their universal applicability in broad contexts and scalability for widespread adoption. The core utility of this system lies in modeling intestinal diseases (e.g., inflammatory bowel disease) and drug metabolism processes through dynamic microenvironments (Kaden et al., 2025; Nguyen et al., 2024), with applications in both basic research and clinical practice; particularly in oncology, gut-on-a-chip platforms can establish complex colorectal cancer microenvironment models (Lee et al., 2025), encompassing the replication of the intestinal TME, for analyzing mechanisms of cancer cell infiltration and metastasis, thereby assessing the efficacy and toxicity of therapeutic compounds, ultimately providing a platform for high-throughput prescreening in cancer therapy and an alternative to animal models for metastasis research.

Organotypic explant cultures are defined as three-dimensional (3D) cultures of intact tissue or organ fragments (He and Deng, 2022) directly obtained from an organism, Their principle relies on optimized culture conditions (e.g., air-liquid interface culture, specialized scaffolds, or matrices) (Zhao Y. et al., 2022) to preserve the spatial architecture of the tissue and intercellular signaling networks (Steindl and Valiente, 2025), thereby simulating physiological or pathological conditions *in vivo*. In cancer research, a key advantage of this model is its faithful preservation of tumor heterogeneity (LeSavage et al., 2022), making it valuable for exploring tumor biology. Limitations include a relatively short culture period that hinders modeling of long-term dynamic processes, technically demanding procedures, and significant challenges in standardization. This model helps reduce the scale of animal experimentation, particularly in cancer pharmacology, by enabling simultaneous assessment of drug effects on both tumor tissue and matched normal tissue to evaluate selective toxicity. It provides a platform for developing targeted therapies and combination immunotherapy strategies.

## 3 Technical advantages and applications of organoids

### 3.1 Unique advantages of organoids in microbiome research

As a three-dimensional (3D) *in vitro* culture system (Kim et al., 2022), organoids can highly simulate the structure, function, and genetic characteristics of human organs, providing an unprecedented platform for studying host–microbe interactions. Compared to traditional models, patient-derived organoids (PDOs) preserve intra- and inter-tumor heterogeneity (Jin et al., 2025) and accurately recapitulate the cellular interaction networks within the original TME. Particularly in gastrointestinal cancer research, organoid technology has demonstrated the ability to recreate key pathological features of tumorigenesis and progression (Idris et al., 2021; Kan et al., 2025), including dynamic interactions between microbes and epithelial cells. Recent studies have found that by integrating artificial intelligence technology with low-biomass microbiome analysis (Han et al., 2021), organoids can reveal intricate interaction mechanisms between microbes and cancer cells. We consider that the evidence for organoids in the simulation of TME and short-term drug response prediction is credible; however, they remain in the proof-of-concept stage regarding dynamic host–microbe interactions and the reconstruction of complex microsystems. Particular caution is warranted: while AI technology can enhance the depth of analysis, the conclusions it infers require dual validation through organoid experiments and clinical data, so as to avoid overinterpreting algorithmic correlations as causal mechanisms.

### 3.2 Comparison with 2D culture systems: microenvironment modeling capability

Two-dimensional (2D) culture systems exhibit significant limitations in modeling the TME, whereas organoids leverage three-dimensional architecture to better recapitulate the composition and organization of the extracellular matrix (ECM) (Jung et al., 2022; Li K. et al., 2025). The development of air-liquid interface (ALI) organoid culture systems (Atanasova et al., 2023) has enabled the establishment of more physiologically relevant models from colorectal cancer patient tissues. Compared to 2D cultures, organoids can maintain the microenvironmental conditions required for long-term co-culture of tumor cells and microorganisms, encompassing critical parameters such as oxygen gradients, nutrient distribution, and mechanical stresses (Kumarasamy et al., 2024). Notably, intestinal organoids have successfully simulated molecular and microenvironmental characteristics of metastatic tumors (Atanasova et al., 2023), a feat unattainable with conventional monolayer cultures. We recognize that organoids are superior to 2D systems, yet they are not without limitations: the full recapitulation of the *in vivo* TME remains a persistent challenge, particularly in terms of standardization and cost, which pose bottlenecks.

### 3.3 Comparison with organ-on-a-Chip Systems: balancing throughput and complexity

Although organ-on-a-chip technology can simulate dynamic microenvironments and tissue-tissue interactions in tumor pathophysiology (Saha et al., 2021; Maity et al., 2025), its limited throughput and operational complexity (Azimian Zavareh et al., 2022) constrain large-scale applications. Organoids, while maintaining sufficient complexity, are better suited for high-throughput drug screening and personalized medicine research (Zhu et al., 2023; Si et al., 2025). Microfluidic chip-integrated organoid systems (Organoid-chip) (Fang et al., 2021) represent a convergence of both technologies’ advantages, enabling the recreation of tissue-specific microenvironments while enhancing experimental throughput and operational feasibility. Particularly in non-small cell lung cancer research (Choi et al., 2023), organoid-immune cell co-culture systems have enabled the observation of dynamic changes in the TME *in vitro*. We argue that balancing experimental complexity and practicality is a key driving factor for model selection. Organ-on-a-chip systems are highly reliable in simulating physiological dynamics such as hypoxia and fluid shear stress, making them well-suited for in-depth mechanistic exploration. Organoids, on the other hand, possess greater advantages in patient-specific modeling and screening efficiency, rendering them particularly suitable for optimizing personalized treatment of non-small cell lung cancer (NSCLC). Integrated systems (organoid-chip) represent an emerging trend; however, their value remains in the preliminary validation stage.

## 4 Analysis of host–microbe interactions in the TME using organoids

### 4.1 Organoid evidence of microbial dysbiosis driving carcinogenesis

#### 4.1.1 Mechanistic evidence for intestinal microbial dysbiosis promoting carcinogenesis by driving chronic inflammation

Intestinal organoid models provide a crucial experimental platform for deciphering chronic inflammation induced by microbial dysbiosis and subsequent carcinogenesis. By remodeling the tumor microenvironment, intestinal microbiota dysbiosis greatly accelerates the infiltration of neutrophils into CRC tissues, thereby leading to mucosal immune dysfunction, stromal cell transformation, and EMT (Li Q. et al., 2025; Tang et al., 2025). This reprogramming of the immune microenvironment enables cancer cells to evade immune surveillance. Disordered microbial communities (e.g., enriched specific pathogenic bacteria) can continuously activate pro-inflammatory signaling pathways (Eiman et al., 2025), resulting in the development of local intestinal and systemic chronic inflammatory states. For example, in mice with TNF receptor deficiency, intestinal organoid experiments have confirmed that TNFR1/2 is targeted to Toll-like receptor 5 (TLR5)-positive Paneth cells and dendritic cells, synergistically contributing to the development of spontaneous ileitis and microbial dysbiosis (Wahida et al., 2021). Notably, the co-culture model of intestinal organoids with dorsal root ganglion (DRG) neurons further demonstrates that inflammation-induced microbial metabolites impair the growth and metabolic functions of colonic organoids while enhancing neuronal excitability; through the release of mediators such as c-Fos and calcitonin gene-related peptide (CGRP), this forms a pro-carcinogenic “microbiota-epithelium-nerve” crosstalk network (Margiotta et al., 2025). Current studies mostly focus on single bacterial species or a limited number of metabolites (Smet et al., 2022; Rivas-Domínguez et al., 2021), and the analysis of the interaction between the overall functional redundancy of microbial communities and the host genetic background remains insufficient. We contend that existing evidence has sufficiently demonstrated the mechanism by which microbial dysbiosis promotes carcinogenesis via inflammatory signaling, whereas evidence related to neural modulation and holistic community interactions remains in the preliminary exploration stage.

#### 4.1.2 Direct evidence of microbial dysbiosis mediating genotoxic effects and bottlenecks in clinical translation

Intestinal organoids exhibit unique advantages in elucidating the mechanisms by which microorganisms exert direct genotoxic carcinogenic effects. Using 3D bioprinted human liver organoids, researchers have verified that bacterial metabolites such as lansoprazole chloride (Lanchlor) can induce significant formation of DNA adducts in organoids, and confirmed their genotoxic damage effects via the comet assay, thereby providing a standardized platform for environmental carcinogen screening (Li et al., 2024). More importantly, the specific mutation signatures (e.g., characteristic base substitution patterns) induced by pks + *Escherichia coli* in intestinal organoids are highly consistent with the genomic mutation profiles of colorectal cancer patients (Rosendahl Huber et al., 2021; Thomas, 2025). This directly demonstrates the ability of specific bacterial strains to induce carcinogenic mutations. This genotoxicity can be achieved through multiple pathways, including microbial metabolites (e.g., secondary bile acids) inducing DNA oxidative damage and chromosomal aberrations (Fu et al., 2021; Yoon et al., 2025). It also involves the continuous accumulation of reactive oxygen species (ROS) in the chronic inflammatory microenvironment, which leads to genomic instability (Xia et al., 2024). Current studies have obvious limitations: most organoid models still rely on the inoculation of single bacterial strains or simplified microbial communities, making it difficult to simulate the complex *in vivo* microbial ecology (Li et al., 2024; Hou et al., 2022). The difficulty in translating organoid-based genotoxicity detection results into clinical practice stems from the complexity of metabolic pathways in the human body and the heterogeneity of microbiota-host interactions among individuals. In organoids, KRAS-mutated cancer cells exhibit chemoresistance (van de Haar et al., 2023). This suggests that microbial dysbiosis may indirectly affect the process of carcinogenesis by altering the threshold of cellular response to genotoxicity. This mechanism requires further verification in organoid models integrated with multi-omics approaches. We argue that intestinal organoids provide an irreplaceable targeted verification platform for the direct genotoxicity of microorganisms; however, they have limitations in recapitulating the ecological complexity of microbial communities and host–microbe interactions. This results in evidence for indirect carcinogenic mechanisms (e.g., metabolic remodeling, immune evasion) still remaining in the preliminary stage. In the future, it will be necessary to enhance translational value by means of multicellular co-culture and organoid-animal model coupling.

### 4.2 Organoid-based validation of tumor-specific microbial features

Organoid platforms provide a key technological foundation for deciphering the spatial and functional heterogeneity of the tumor microbiome (Kan et al., 2025; Poletti et al., 2021), Microfluidic organoid-chips simulate microbial colonization within hypoxic niches, demonstrating that hypoxic microenvironments enrich drug-resistant microbiota through selection pressure, thereby contributing to chemotherapy resistance. In PDO models, low alpha-diversity microbial communities show significant correlation with loss of MHC-I expression (Yáñez-Bartolomé et al., 2023; Zhou et al., 2023), leading to reduced CD8 + T cell infiltration and immunotherapy resistance (Wang et al., 2023). Notably, while organoids can recapitulate tumor-type specificity reflected in microbial composition, they remain limited in simulating multi-species synergistic effects, particularly in maintaining the viability of obligate anaerobes over extended periods. We contend that the value of organoids in simulating tumor heterogeneity and single microbe-host interactions (e.g., hypoxia-induced drug resistance) has been fully validated. However, these mechanisms have mostly been explored through correlational studies, rather than direct causal validation. Its ability to simulate multi-species synergistic effects (e.g., maintenance of anaerobic bacteria) remains in the preliminary exploration stage. And there are significant technical controversies associated with this capability. In the future, interdisciplinary innovation will be required to enhance the integrity of such models.

## 5 Cancer applications: from mechanistic insights to clinical translation

### 5.1 Key technological breakthroughs of organoids in cancer microbiome research

#### 5.1.1 Precision co-culture techniques break the confines of traditional models

As a three-dimensional model, organoids address the limitations of traditional two-dimensional cell cultures in simulating tissue architecture and spatial host–microbe interactions. Through direct microinjection of gut microbiota (or specific pathogens) into the lumen of hollow organoids and the establishment of co-culture systems (Verhulsel et al., 2021; Wang et al., 2024), this technology enables the simulation of physical contact and biomolecular exchange between host cells and microbes. More advanced organoid-on-a-chip models incorporate microfluidic technology (Valiei et al., 2023), allowing better recapitulation of physiological dynamics such as intestinal fluid flow and shear stress, thereby making the interaction process more representative of the *in vivo* environment. In the technological breakthrough of “immune-gut microbiota co-culture systems,” organoids utilize a three-dimensional co-culture environment to integrate intestinal organoids, gut microbiota, and associated immune cells (Shek et al., 2021) (e.g., T cells or NK cells). By employing microfluidic platforms and microinjection techniques (Liu et al., 2024), this system achieves the goal of simulating host–microbe interactions within the TME *in vitro*. It overcomes the limitations of traditional two-dimensional models and provides physiologically relevant insights into how gut microbiota modulate immunity in colorectal cancer (Golpour et al., 2023), such as microbiota-mediated mechanisms of T cell activation and NK cell cytotoxicity influencing tumor progression, immune evasion, and treatment response. Furthermore, it serves as an experimental platform for developing personalized immunotherapies (Wu et al., 2025) (e.g., microbiota-targeted modulation). However, the system still faces core limitations, including challenges in reconstituting the full spectrum of complex immune cells, standardization issues, and high costs (Chen et al., 2022; Kriaa et al., 2024). We think that 3D organoid models can highly simulate the physical interactions, biomolecular exchange, and basic immune activation between hosts and microorganisms. And their physiological relevance is significantly better than that of 2D models. The bidirectional regulatory role of microorganisms in the tumor immune microenvironment still requires more evidence to confirm. Furthermore, the dynamic reconstruction of complex immune cell networks remains a technical bottleneck.

#### 5.1.2 Multi-omics integration platforms

Research on host-gut microbiota interactions within the TME has achieved significant advancements through the integration of organoids and multi-omics platforms (Kim et al., 2022; Coxon et al., 2025), enabling researchers to accurately simulate the complex characteristics of the colorectal cancer microenvironment *in vitro* and analyze dynamic interaction mechanisms between gut microbiota and host cells. By integrating data from genomics, transcriptomics, metabolomics, and microbiome studies (Liu B. et al., 2025; Zhang et al., 2023), this approach reveals potential molecular drivers and therapeutic targets. The combination of organoids with single-cell sequencing and metabolomics (Smirnov et al., 2023; Zhao H. et al., 2022) elucidates how microbiota modulate the *β*-catenin signaling pathway and cellular self-renewal processes under tumor heterogeneity. High-throughput platforms such as bioreactors, combined with machine learning algorithms (Wang Q. et al., 2025), facilitate large-scale drug sensitivity testing. However, multi-omics integration still faces significant challenges, including reduced reproducibility (Su et al., 2025) due to high data heterogeneity and noise, and difficulties in causal inference (Metwaly et al., 2022) (e.g., challenges in determining whether microbiome changes are drivers or consequences of disease) and insufficient standardization (Babu and Snyder, 2023). These limitations impact its broad applicability in clinical translation, but predictive models integrating artificial intelligence are expected to enhance data interpretation and advance the potential for personalized therapy applications. We affirm that organoid-like models can highly accurately simulate tumor-microbe interactions during multi-omics integration and have also become effective tools for drug screening. Their clinical translational value is hampered by data noise, ambiguous causal logic, and a lack of standardization. The “driver or bystander” role of microorganisms still needs to be further verified through spatial multi-omics and longitudinal studies (see Table 2).

**Table 2:** Application and validation of organoids in tumor microbiome research.

Feature dimension	Validation contribution of organoids	Key technologies	Limitations
Compositional features	Organoid co-culture systems recapitulate tumor-specific microbial colonization (e.g., *Fusobacterium nucleatum* enrichment in CRC organoids). Confirmed that low Alpha Diversity environments promote the dominance of carcinogenic strains	Microbial injection, Spatial transcriptomics	Difficulty in simulating in vivo hypoxic/acidotic selective pressure on microbiota
Spatial heterogeneity	Organoid-on-chip models achieve simulation of microbial gradient distribution (e.g., core vs. edge microbial differences in tumors). Revealed that local metabolite concentration gradients drive cancer cell migration	Microfluidic gradient generation, Fluorescence labeling and tracking	Lack of vascular system affecting systemic metabolite distribution
Dynamic evolution	Long-term cultured organoids capture microbiota changes during cancer progression. Discovered shift from early pro-inflammatory microbiota (e.g., *E. coli*) to late immunosuppressive microbiota (e.g., *Bacteroides*)	Time-lapse imaging, Temporal metagenomic analysis	In vitro culture period (<8 weeks) insufficient to simulate multi-year carcinogenesis
Host interaction specificity	Patient-derived organoids (PDOs) retain individualized microbiota-host interaction features. APOBEC-mutant tumor organoids specifically recruit oral-origin microbiota	Single-cell sequencing, Host-microbe co-culture	Inter-individual variability challenging reproducibility

#### 5.1.3 Bidirectional regulatory mechanisms of microbial metabolites

##### 5.1.3.1 Carcinogenic effects

The carcinogenic effects of intestinal microbial metabolites are mainly due to the disruption of immune surveillance caused by their abnormal production, with specific mechanisms involving the induction of genetic mutations and alterations to the tumor microenvironment. When the production of metabolites is abnormal, the host immune system fails to recognize and eliminate tumor cells in the early stages of carcinogenesis, leading to tumor progression, and this phenomenon is particularly prominent in colon cancer (Liu et al., 2023). Key metabolites such as short-chain fatty acids (SCFAs), polyamines, and secondary bile acids directly participate in tumorigenesis by regulating immune responses; they promote the occurrence of gene mutations in epithelial cells or impair the ability of immune cells to clear abnormal cells (Liu et al., 2023; Hanus et al., 2021). This pro-carcinogenic effect does not exist in isolation: bacteria such as Fusobacterium alter inflammatory signals in the microenvironment, leading to the emergence of immunosuppressive states, yet the specific molecular pathways remain not fully elucidated. This indicates that there are still inadequacies in mechanistic research—for example, the causal link between intestinal microbiota dysbiosis and colorectal cancer (CRC) risk is currently unclear (Duan et al., 2024; Fan et al., 2025). The application of intestinal organoid models in Lynch syndrome has shown potential, enabling the evaluation of how epigenetic modulation (e.g., EZH2-mediated immune gene modification) drives carcinogenesis (Bowen et al., 2025). However, it is still limited by this constraint: organoids cannot fully simulate the complexity of *in vivo* host–microbe interactions. Additionally, the dual effects of different metabolites (e.g., certain SCFAs possess both anti-tumor capacity and pro-tumor effects) constitute a major research barrier, and rigorous experimental design is urgently needed to resolve this contradiction.

##### 5.1.3.2 Anti-carcinogenic effects

In the context of cancer resistance, intestinal microbial metabolites remodel the tumor microenvironment through immune regulation, enhancing host defense capabilities and anti-tumor efficacy; however, this process is limited by individual differences and therapeutic variability. Specific metabolites, such as microbial tryptophan catabolites, can activate immune control mechanisms (Jaye et al., 2022), inhibit the progression of obesity-related cancers, and remodel the microenvironment to facilitate the clearance of abnormal cells. These metabolites enhance the anti-tumor efficacy of chemotherapy, radiotherapy, and immunotherapy by regulating immune cell functions (e.g., enhancing T cell activity) (Song et al., 2025; Wang Y. et al., 2025; Yang et al., 2023). In model systems, they exhibit effects of reducing treatment-related side effects and improving prognosis. From a critical perspective, clinical application faces significant challenges: variations in the individual microbiome lead to inconsistent metabolite production, which affects the prediction of therapeutic efficacy (Nazir et al., 2025). Even though engineered bacteria strategies (e.g., designing degrading enzymes to target specific metabolites) have shown potential in mouse models (Wang S. et al., 2022), their translation to humans often fails due to the complex nature of host-immune interactions. By screening probiotic or prebiotic combinations, intestinal organoids can assist in testing the anti-carcinogenic mechanisms of microbe-targeted strategies. However, existing models struggle to capture the full-spectrum effects of metabolites in systemic immune regulation, highlighting the need to integrate high-throughput metabolomics to achieve precision medicine-guided optimization.

##### 5.1.3.3 Immune regulation

The immune regulatory effects of intestinal microbial metabolites manifest as dual regulation of the tumor immune environment; their mechanisms involve the dynamic balance between receptor activation and cytokines, yet the underlying regulatory networks remain highly controversial. Metabolites such as indole-3-acetic acid (IAA) can activate the aryl hydrocarbon receptor (AhR), leading to differential expression of cytokines and changes in immune cell composition, thereby providing support for the regulation of anti-cancer immune checkpoints (Yang et al., 2023; Vaaben et al., 2025). Within the tumor microenvironment, these metabolites can remodel innate and adaptive immune responses (Lu et al., 2022; Kovtonyuk and McCoy, 2023) and influence the efficacy of immunotherapy. At the same time, the consumption of key metabolites by microorganisms (e.g., immune suppression mediated by regulatory T cells (Treg)) (Meza-Perez et al., 2024) may counteract their positive effects, resulting in impairments in immune function. Intestinal organoids in ex vivo models can help simulate the regulation of immune cell dynamics by metabolites; however, critical analysis reveals that the molecular pathways of epigenetic regulation have not yet been fully elucidated (Duan et al., 2024). The complexity of host–microbe interactions often leads to biases in data interpretation (Duan et al., 2024; Li et al., 2022). In the future, it will be necessary to use organoids to integrate multi-omics data, clarify the dual balance of immune regulation, and thereby overcome the bottleneck in precision intervention. We contend that gut microbial metabolites regulate the tumor immune microenvironment and therapeutic responses via receptor-mediated pathways, and their role has received direct experimental support in organ-specific organoid models (e.g., for colon cancer). This evidence is reliable and holds clinical translational potential. However, current organoid technology still has limitations in simulating stromal-immune cell interaction networks. For instance, the dynamic integration of fibroblasts and myeloid cells has not yet been standardized. This may lead to an underestimation of the complexity of microenvironmental pathways.

#### 5.1.4 Analysis of key signaling pathways using organoids

**Figure 2:**
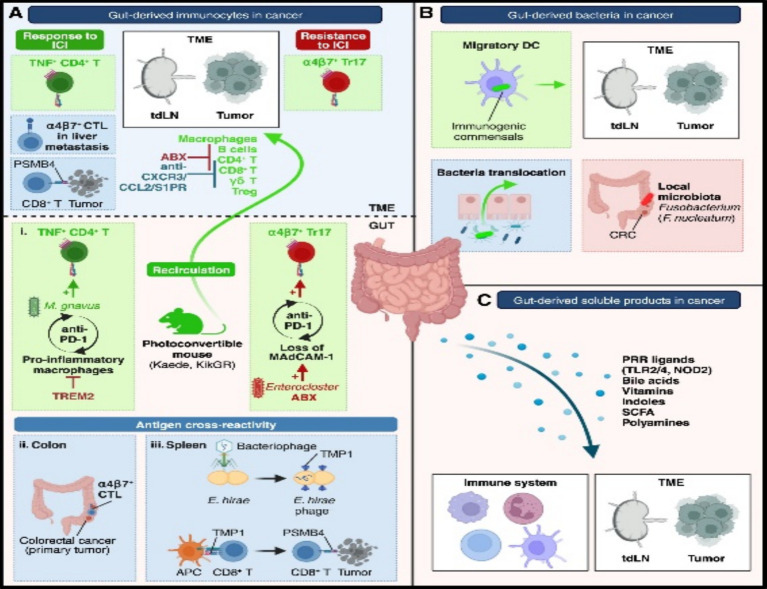
Mechanisms of gut influence on the TME. **(A)** Gut-derived immunocytes migrate to the TME under microbiota influence. **(B)** Gut bacteria can translocate to the TME via dendritic cells or barrier leakage. **(C)** Soluble gut-derived factors modulate cancer–immunity crosstalk. ABX, antibiotics; CRC, colorectal cancer; CTL, cytotoxic T cells; DCs, dendritic cells; TME, TME; Treg, regulatory T cells. Adapted with permission from Pich et al. (2025). Copyright 2024 Immunity.

Organoids, as three-dimensional (3D) culture systems, recapitulate the structural, functional, and genetic characteristics of human organs—including epithelial, immune, and microbial components within tumor tissues—thereby providing a highly controlled environment for studying key signaling pathways regulated by the microbiota. For example, studies using organoids have demonstrated that microbiota-derived short-chain fatty acids (SCFAs) activate host signaling pathways (e.g., G protein-coupled receptor pathways) (Xu J. et al., 2022), modulating the tumor immune microenvironment and influencing cancer progression. These metabolites directly act on epithelial cells in organoids, revealing their role in promoting inflammation and carcinogenesis (Boesch et al., 2022). Furthermore, organoids enable in-depth dissection of inflammation-related pathways—such as the TLR4-NF-κB-NLRP3 inflammasome axis (Sheng et al., 2021; Xie and Liu, 2024)—and their roles in driving chronic inflammation and immunosuppression within the TME during microbial dysbiosis. By simulating bacterial interactions, organoids demonstrate how microbiota imbalance amplifies the activation of these pathways, leading to uncontrolled cell proliferation and therapy resistance. Additionally, organoids help elucidate how the microbiota regulates other signaling pathways (Zhang X. Y. et al., 2024; Wu et al., 2023) (e.g., PI3K-AKT and TGFβ signaling) via the intratumoral microbiome, which are critically involved in tumor cell metabolism, metastasis, and response to immunotherapy. For instance, studies combining patient-derived organoids with microbiota transplantation have revealed that microbial metabolites (e.g., lipopolysaccharide or bacterial toxins) indirectly affect immune checkpoint pathways (e.g., PD-1/PD-L1) by remodeling the stromal components of the TME (Si et al., 2025; de Castilhos et al., 2024), providing an experimental foundation for designing microbiota-targeted therapies. Collectively, organoids have revolutionized cancer research by elucidating key signaling pathways, bridging the limitations of traditional cell lines and animal models, offering human-relevant simulations, and advancing our understanding of dynamic host-microbiota collaborations within the TME. We contend that gut microbial metabolites regulate the tumor immune microenvironment and therapeutic responses through receptor-mediated pathways, and their role has received direct experimental support in organ-specific organoid models (e.g., models for colon cancer). This evidence is reliable and holds clinical translational potential. However, current organoid technology still has shortcomings in simulating stromal-immune cell interaction networks. For example, the dynamic integration of fibroblasts and myeloid cells has not yet been standardized. This may lead to an underestimation of the complexity of microenvironmental pathways (Figure 2).

### 5.2 Clinical translation scenarios and challenges

#### 5.2.1 Personalized drug screening

**Figure 3:**
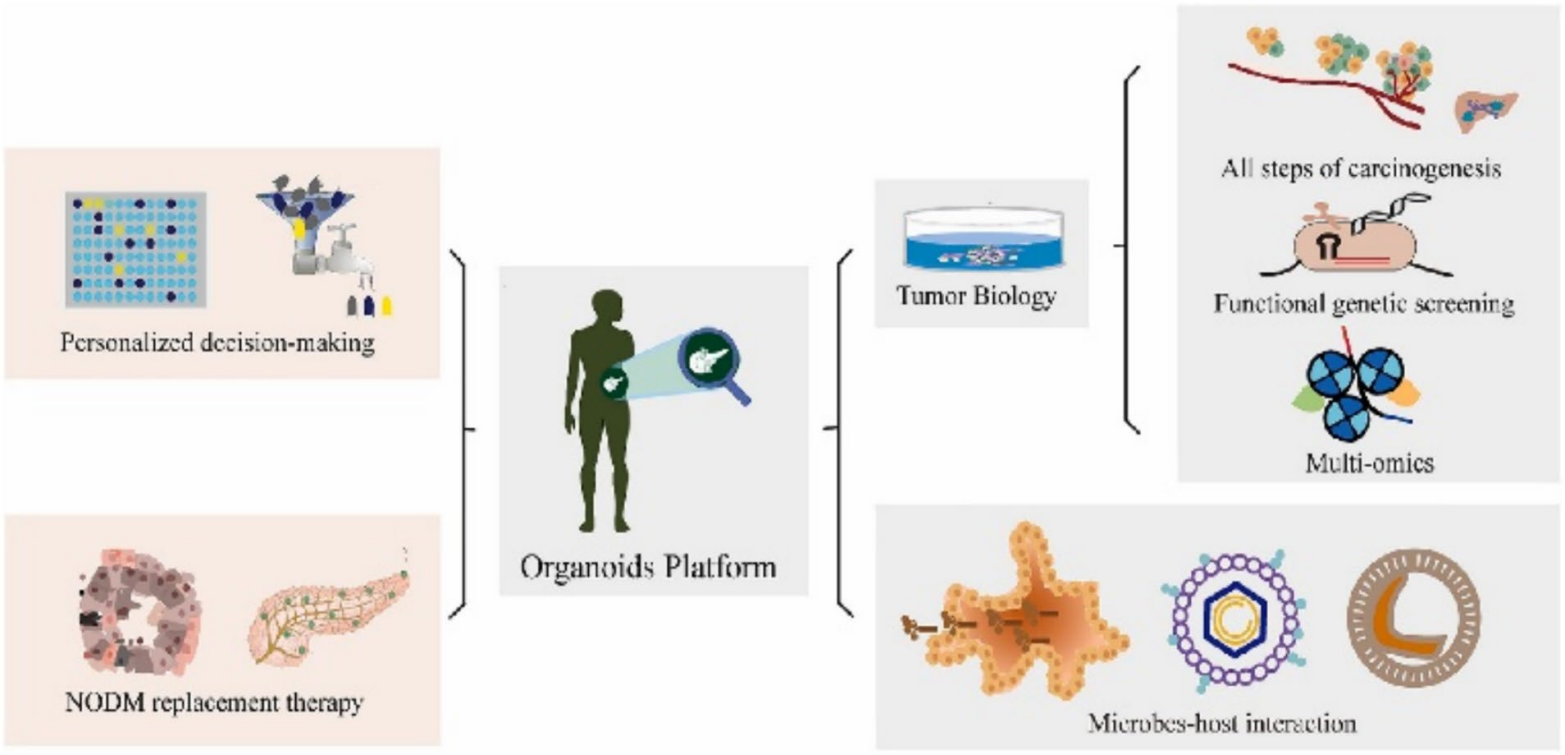
Organoids serve as a transformative platform, bridging the gap between basic biological research and clinical practice by facilitating bidirectional translation. Adapted with permission from Wang Y. et al. (2025). Copyright 2023 Cancer Lett.

Organoids serve as *“patient-specific TME-mimicking models”* that retain the genetic and phenotypic attributes of the original tumors (Liu et al., 2021; Zitvogel et al., 2024), providing a platform for evaluating individual patient responses to chemotherapy and targeted therapies (Zhou et al., 2025). By exposing patient-derived organoids to various drug conditions (Yan et al., 2024), treatment efficacy can be predicted, thereby reducing the risk of ineffective therapies. A major challenge is that current organoids often lack a complete tumor immune microenvironment (Song et al., 2024), limiting their ability to accurately simulate the impact of microbial metabolites (e.g., short-chain fatty acids, SCFAs) on drug metabolism or immunotherapy responses. Proposed solutions include developing immune-organoid co-culture models by incorporating autologous immune cells or engineering organoids to simulate the tumor immune microenvironment (TIME), alongside advanced microbiota-host co-culture systems (Magré et al., 2023), aiming to reconstitute microbiota-mediated modulation of drug sensitivity *in vitro*. We contend that the mechanisms underlying the recapitulation of tumor biology and drug sensitivity have received relatively sufficient verification. However, the issue of the lack of immune microenvironment and its remedial strategies are in the preliminary experimental stage. There are controversial issues regarding reproducibility and clinical translation (Figure 3).

#### 5.2.2 Optimization of immunotherapy

In this context, organoids serve as a platform (Weng et al., 2023) to investigate how specific microbiota or their metabolites reshape the local tumor immune response (Pich et al., 2025), and to conduct screening of immunotherapy drugs (Grönholm et al., 2021). However, a key challenge is that current organoids struggle to fully recapitulate the complex *in vivo* immune regulatory network and the systemic microbiota-immune axis. Immune cells cultured in vitro may become functionally exhausted, and organoids often fail to maintain the physical and biochemical integrity of the host mucosal barrier (Hanus et al., 2021), which is a critical interface for microbiota-immune interactions. Proposed solutions include developing microfluidic organ-on-a-chip technology (Grönholm et al., 2021) to integrate immune and microbial components, and employing genetic engineering to modify organoids, thereby enabling more precise simulation of specific immune pathways (Figure 3).

#### 5.2.3 Early disease diagnosis and mechanism studies

##### 5.2.3.1 Microbial biomarkers

Intestinal organoids provide an innovative platform for the discovery of microbial biomarkers in early tumor diagnosis, and elucidate the interaction mechanisms between the intestinal microbiota and tumors by establishing highly physiologically relevant models. A dual-channel system constructed using intestinal organoids differentiated from human induced pluripotent stem cells (hiPSCs) can incorporate patients’ fecal samples for multi-omics analysis, thereby enabling the identification of epithelium-specific biomarkers and key microbial factors associated with the clinical outcomes of melanoma (Ballerini et al., 2025). The advantages of this approach lie in the ability of organoids to more accurately simulate the intestinal microenvironment, which overcomes the limitations of traditional 2D models. It supports the study of host–microbe interactions under controlled conditions and provides an efficient avenue for the high-throughput screening of tumor-associated microbial biomarkers. Organoid models can recapitulate patients’ genetic characteristics (Xiang et al., 2024), enabling more personalized screening for specific tumor markers and thus demonstrating potential in the identification of diagnostic biomarkers and therapeutic targets. Notable limitations of the organoid system remain: it lacks immune components and the *in vivo* dynamic environment (Özkan et al., 2024; Bouffi et al., 2023), making it difficult to fully recapitulate gut-immune crosstalk or long-term microbial effects. This is likely to affect the clinical translatability of microbial biomarkers. Although organoids can identify associations between changes in microbial communities and disease progression, their predictive capacity is limited in the absence of immune regulation and mechanical stress (e.g., peristalsis) (Özkan et al., 2024), which may lead to biases in the in vivo validation of biomarkers.

We contend that (organoids) possess irreplaceable tool value in the process of revealing microbe-host interaction mechanisms and conducting high-throughput preliminary screening of biomarkers, and their experimental controllability far exceeds that of clinical sample analysis. All organoid-related findings must undergo secondary validation using immune co-culture models or *in vivo* animal validation; otherwise, there may be an overestimation of the clinical translatability of biomarkers, as these findings have not yet passed the “stress tests” of immune regulation and dynamic physiological environments.

##### 5.2.3.2 Metabolite biomarkers

Regarding metabolite biomarkers, intestinal organoids have provided key clues for early tumor diagnosis by integrating metabolomics techniques, particularly when elucidating tumor-related metabolic reprogramming mechanisms. Relevant studies have confirmed that organoid models can be used to detect intestinal microbial disorders induced by cold stress and their association with liver injury; by analyzing changes in glycerophospholipid metabolism, ABC transporters, and purine metabolism pathways, the correlation between specific microbial taxa and metabolite biomarkers can be identified (Liu A. et al., 2025). The advantage of this approach lies in the high-throughput culture capability of organoids (Xiang et al., 2024), which supports real-time monitoring of metabolic changes. When combined with untargeted metabolomics approaches, it can sensitively detect subtle metabolic abnormalities (e.g., metabolic perturbations caused by toxic exposure) (Xuan et al., 2024), providing a high-sensitivity tool for the discovery of tumor markers. Organoid models are also suitable for studying the effects of dietary or pharmaceutical interventions on metabolite markers. For example, in simulating nutrient metabolism and toxicity analysis, they facilitate the identification of relevant targets for metabolic diseases such as diabetes, thereby providing support for the development of anti-tumor drugs (Nie et al., 2023). Organoids have limitations in simulating metabolite dynamics: they cannot effectively recapitulate in vivo mechanical forces (e.g., digestive fluids) or long-term microbial ecological balance (Özkan et al., 2024), and may overestimate the stability of metabolic pathways (Lu et al., 2024). We affirm that the core advantages of organoid metabolic models lie in their high sensitivity and pathological specificity; however, their simplified physiological simulation and inadequate recapitulation of tumor heterogeneity may diminish their predictive value. In terms of the current evidence chain, the mechanisms underlying the interaction between environmental exposure and microorganisms are relatively sufficient, while the response data generated by pharmaceutical interventions require more targeted validation. The modeling of inter-organ metabolic networks remains in the exploratory stage.

## 6 Concluding remarks and outlook

Organoids have undoubtedly revolutionized the research paradigm for investigating the complex relationships between the gut microbiota and the TME (TME). They have established a physiologically relevant research platform that breaks through the barriers of traditional models. Organoids are capable of preserving the patient-specific genetic and phenotypic characteristics while supporting co-cultivation with microbial communities. This attribute provides unprecedented perspectives for in-depth understanding of host–microbe interactions, metabolic regulation, and immune modulation mechanisms within the TME. However, this field is still in its initial stages, and multiple challenges must be addressed to fully tap into the potential of organoid technology.

The integration of multi-omics technologies (such as spatially resolved transcriptomics, metabolomics, and proteomics) with advanced organoid systems is crucial for unraveling the spatiotemporal dynamics of interactions between the gut microbiota and the TME. The development of more complex co-culture systems (incorporating immune cells, stromal components, and neuroendocrine elements) is also essential; only in this way can the systemic interaction processes observed in vivo be recapitulated. The rise of organoid-on-a-chip technology, combined with machine learning-driven analytical approaches, holds the potential to construct predictive models that can accelerate personalized drug screening and the development of therapeutic strategies.

To translate organoid-based research findings into clinically applicable outcomes, it is still necessary to establish standardized experimental protocols, promote the construction of scalable biobanks, and conduct rigorous validation in human cohorts. By bridging the gap between experimental models and human pathophysiological processes, organoid technology is likely to lead the next wave of innovations in the field of precision oncology, providing new avenues for cancer therapy targeting the TME-gut microbiota axis.
